# Linkage of HIV-Infected Infants from Diagnosis to Antiretroviral Therapy Services across the Western Cape, South Africa

**DOI:** 10.1371/journal.pone.0055308

**Published:** 2013-02-06

**Authors:** Nei-Yuan Hsiao, Kathryn Stinson, Landon Myer

**Affiliations:** 1 Division of Virology, University of Cape Town and National Health Laboratory Service, Cape Town, South Africa; 2 Centre for Infectious Diseases Epidemiology and Research, School of Public Health and Family Medicine, University of Cape Town, Cape Town, South Africa; University of Washington, United States of America

## Abstract

**Introduction:**

Early infant diagnosis (EID) of HIV infection is an important service to reduce paediatric morbidity and mortality related to HIV/AIDS. Although South Africa has a national EID programme based on PCR testing, there are no population-wide data on the linkage of infants testing HIV PCR-positive to HIV care and treatment services.

**Methods:**

We conducted a retrospective analysis of all public sector laboratory data from across the Western Cape province between 2005 and 2011. We linked positive HIV PCR results to subsequent HIV viral load testing to determine the proportion of infants who were successfully linked to HIV care.

**Results:**

A total of 83 698 unique infant HIV PCR tests were documented, of which 6322 (8%) were PCR positive. The proportion of PCR-positive children declined from 12% in 2005 to 3% in 2011. Of the children testing PCR-positive, 4105 (65%) had subsequent viral load testing indicating successful linkage to care. The proportion of successfully linked infants increased from 54% in 2005 to 71% in 2010, while the median delay in days to successful linkage decreased from 146 days in 2005 to 33 days in 2010.

**Discussion:**

From 2005 to 2011 there has been a reduction in the proportion of children testing HIV PCR-positive, and an increase in the proportion of infected infants successfully linked to HIV care and treatment, in this setting. However a large proportion of infected infants remain unlinked to antiretroviral therapy services and there is a clear need for interventions to further strengthen EID programmes.

## Introduction

Across sub-Saharan Africa, mother-to-child-transmission (MTCT) of HIV infection remains an ongoing threat to child health. Despite the widespread implementation of PMTCT programmes, an estimated 390,000 infants were newly infected with HIV during 2010 alone [1]. Infant HIV infection often results in rapid HIV disease progression as approximately 50% of vertically-infected infants die in the first year of life [2]. In South Africa, where there were an estimated 40 000 new infant HIV infections during 2010, HIV contributes significantly towards preventable infant mortality [3], [4].

Early identification of perinatally-infected infants and rapid referral for initiation of antiretroviral therapy (ART) is an important intervention to promote child health [5], [6]. Early infant diagnosis (EID) of HIV by polymerase chain reaction (PCR) is routinely used to detect HIV infection in infants. Although EID is an important component of effective PMTCT programmes, HIV PCR testing is relatively expensive, requires specialised laboratory equipment, and is time consuming. This means the testing components of EID is firmly in the domain of centralised specialist laboratories. This in turn makes health systems issues and logistical considerations, such as conducting HIV testing in primary care facilities, transporting specimens to central reference laboratories, and return of results to primary care, a major concern [7], [8].

Across Africa, non-retention of patients and delays in testing and referral are major operational concerns facing EID programmes [9]. Loss to follow-up of infants between HIV testing, the return of results, and referral of infected infants to paediatric ART services has been documented in several settings [10]–[13]. Even when infants are retained throughout these steps, the delays involved in testing and referring infants for ART may be unacceptable given the high mortality observed in infected infants who are not yet on ART [12]–[15]. But while EID services across Africa face important challenges, systems for monitoring EID programmes are not well developed; as a result, there are few population-level data on the performance of EID services in identifying HIV-infected infants and referring them to ART services [16].

In South Africa, there are few data on the performance of EID services in referring infected infants to long-term care. We used routinely collected HIV laboratory data to investigate the overall levels of MTCT in the Western Cape Province, and the performance of the EID service in referring infected infants for ART, between 2005 and 2011.

## Materials and Methods

PMTCT and EID services operate at public sector primary care clinics and hospitals throughout the Western Cape province. HIV PCR testing is used for HIV screening in HIV-exposed infants attending routine postnatal immunization clinics as well as for HIV diagnosis in children who present to hospitals. Prior to 2008, newly diagnosed infants were referred to specialist paediatric infectious disease clinics operated in secondary and tertiary hospitals for ART initiation and follow-up; the provision of ART was based on the 2004 WHO recommendations [17]. Following the results of the Children with HIV Early Antiretroviral Therapy (CHER) study released in 2008, ART initiation for all infected infants became policy across the province, with ART delivered through hospitals as well as a growing number of primary care clinics. Throughout, HIV viral load testing was routinely performed by ART services prior to treatment initiation. This ‘baseline’ viral load serves as a confirmation of the positive HIV PCR result and is a tool for which subsequent treatment efficacy can be measured.

### Study Objective

This study sought to describe the linkage of HIV-infected children to ART care using public sector laboratory testing data.

### Sources of Data

Data for this analysis are all HIV PCR and HIV viral load testing results from public sector health services in the Western Cape Province between January 2005 and July 2011. Data came from the central data warehouse of the National Health Laboratory Service, the sole provider of pathology services for the public health sector in South Africa. The following data were available: patient name and provincial folder number; health facility; type and date of test and test result; and patient date of birth and gender. We identified the first positive HIV PCR result for each child under the age of 2 years, and the first HIV viral load for each child under the age of 5 years, for inclusion in the analysis. Test results related to quality assurance/quality control, and tests of patients enrolled into clinical trials, were excluded.

### Data Analysis

Data were analysed used Stata Version 11.0 (Stata Corporation, College Station, Texas, USA). We used the date of a child’s positive HIV PCR test result as the date of diagnosis, and used the date of the first HIV viral load as the date of first attendance at ART services. HIV PCR and viral load results for individual children were linked using combinations of folder number, name and date of birth. We defined ‘definite’ links as matching identical full surname or folder number in combination with an identical date of birth and ‘probable’ links as matching any combination of partial surname, date of birth and folder number. However, study findings did not differ appreciably between definitions, and the results presented here are based on all ‘probable’ linkages.

The delay in days between the first positive HIV PCR and the first HIV viral load test was used to estimate the delay between a PCR positive test result and a child’s first attendance at an ART clinic. Children who were HIV PCR-positive but did not have a HIV viral load test were considered to have not attended ART services. A specialist hospital was defined as a hospital where a paediatrician with infectious diseases training was present, while the remaining facilities are primary and secondary care facilities administered by doctors and/or nurses without specific paediatric infectious disease training. An urban facility was defined as the site of testing within the greater Cape Town area; the remaining facilities in the province are considered rural facilities.

In analysis, continuous variables were described using medians and interquartile ranges (IQR) while proportions with exact binomial 95% confidence intervals (CI) were calculated for categorical variables. Logistic regression models were used to examine the independent predictors of (i) positive HIV PCR test results, and (ii) the successful referral of infected children to ART services; the results are presented as odds ratios (OR) with 95% CI. Variables in the model were selected based on a priori evidence and findings of descriptive statistics.

### Ethics Statement

The study was approved by the National Health Laboratory Services and the Research Ethics Committee of the Faculty of Heath Sciences at the University of Cape Town.

## Results

A total of 83 698 children less than 2 years of age underwent HIV PCR testing at public sector health care services in the Western Cape between 2005 and 2011. Of these, 6322 (7.6%) tested positive, 76 956 (91.9%) tested negative and 418 (0.5%) of PCR results were equivocal (Table 1). The number of HIV PCR tests almost doubled over time, while the proportion of PCR-positive children declined from 12% in 2005 to 3% in 2011. The median age of first HIV PCR testing was approximately 4 months (IQR 3.1–5 months) during 2005 and decreased to 1.5 months (IQR 1.4–2.1 months) during 2011.

**Table 1:** Early infant diagnosis data, Western Cape, South Africa, 2005–2011.

		HIV PCRpositive	HIV PCR negative	Total	Percent positive	OR (crude)	95%CI
Year	2005	1057	7594	8651	12%	1.0	(reference)
	2006	861	6193	7054	12%	1.00	0.91–1.10
	2007	1170	11416	12586	9%	0.74	0.67–0.80
	2008	1097	13279	14376	8%	0.59	0.54–0.65
	2009	1035	14640	15675	7%	0.51	0.46–0.56
	2010	793	14931	15724	5%	0.38	0.35–0.42
	2011*	311	8903	9214	3%	0.25	0.22–0.29
Sex	Female	3215	36648	39863	8%	1.0	(reference)
	Male	2848	36289	39137	7%	0.89	0.85–0.94
Facility	Primary care facility	3757	68605	72362	5%	1.0	(reference)
	Specialist Hospitals	2567	8351	10918	24%	5.61	5.31–5.93
Urban facilities	Rural	1953	22480	24433	8%	1.0	(reference)
	Urban	4372	54494	58866	7%	0.92	0.87–0.98
Age at time of PCR	≤2 months	1999	41151	43150	5%	1.0	(reference)
	>2 months	4326	35823	40149	11%	2.49	2.35–2.62

Factors associated with HIV PCR results amongst HIV-exposed children less than two years of age tested for the first time at public sector health facilities in the Western Cape Province of South Africa, January 2005 and July 2011.

* For 2011, results are from January to June.

In 2005, 303 health facilities conducted HIV PCR testing, increasing annually to 341 facilities in 2010. Thirteen percent of PCR testing came from hospitals with specialist paediatric services and 69% of PCR tests were from urban facilities around Cape Town. No significant changes in the proportions of specialist hospital and urban PCR testing were observed over the study period; however infants tested at specialist hospitals were much more likely to be PCR positive (p<0.001). Just over half of infants (51%) were tested under 3 months of age, the age targeted for the current EID programme.

During the same period, 11 653 first HIV viral load tests were carried out in children <5 years of age at public sector health care facilities. The median viral load of the samples with detectible HIV was 150 390 copies (IQR 12 975–990 000 copies/ml). The number of facilities that conducted HIV viral load testing rose from 91 in 2005 to 220 in 2010 (Table 2). Sixty-nine percent of the first time paediatric HIV viral load tests was performed in an urban facility; this proportion declined from 73% in 2005 to 60% in 2010.

**Table 2:** Changes in early infant diagnosis, HIV viral load testing, and linkages to care over time.

		Year					
		2005	2006	2007	2008	2009	2010
Number of facilities testing	HIV PCR	271	276	312	330	322	343
	HIV Viral load	91	133	149	203	216	220
PCR tests done		8653	7065	12603	14416	15743	15922
Facility	Specialist Hospitals	1242	1430	1793	1941	1839	1761
	Primary care facility	7411	5634	10812	12477	13904	14161
	*% from specialist care*	*14*	*20*	*14*	*14*	*12*	*11*
Facilities	Urban	5752	4206	8997	10265	11159	11120
	Rural	2901	2858	3605	4153	4584	4802
	*% Urban samples*	*67*	*60*	*71*	*71*	*71*	*70*
Referral to care	Referred	576	483	662	758	703	566
	Not referred	481	378	508	337	332	227
	*% referred*	*54*	*56*	*57*	*69*	*68*	*71*

Changes in HIV PCR/VL testing facilities and linkage to care for HIV PCR positive children less than two years of age attending public sector health facilities in the Western Cape Province of South Africa, January 2005 to July 2010.

### Linkage of PCR-positive Children to ART Services

Using the ‘definite’ matching criteria, we found 3414 of 6322 (54%) children with first positive HIV PCR who also had a HIV viral load conducted prior to ART initiation and were thus considered successfully linked to care. Under the ‘probable’ matching criteria we were able to match a further 691 PCR-positive children to HIV viral load results, resulting in a total of 4105 children (65%) who were HIV PCR-positive and had laboratory evidence of attending ART services.

The distribution of PCR-positive children who were and were not linked to ART services is shown in Table 3. Over the period, the proportion of linked children increased from 54% in 2005 to 71% in 2010. The linkage rate of children diagnosed HIV PCR-positive at specialist hospitals (69%) was substantially higher than at primary care centres (58%); despite contributing 13% of all PCR tests requested, the specialist hospitals accounted for 45% of all children successfully linked to ART services. In particular, Cape Town’s largest paediatric hospital contributed 25% of all children linked to ART services in the province. Related to this, successful linkage of PCR-positive children to ART services was significantly more likely at urban health facilities (68%) compared to rural facilities (50%). This difference appeared independent of urban-rural variation (OR 1.89, 95%CI 1.68–2.14). In addition, children older than 2 months of age appeared to be less likely to be linked (OR 0.73, 95% CI 0.64–0.81) compared to younger infants; this association persisted after adjusting for potential confounding variables such as the year of testing and facility of testing (OR 0.82, 95% CI 0.72–0.92).

**Table 3:** Proportion of HIV PCR-positive infants linked to HIV treatment services.

		HIV PCR-positive children with linked VL					
		Linked	Not linked	Total	Percent	OR (crude)	95%CI
Year	2005	576	481	1057	54%	1.0	(reference)
	2006	483	378	861	56%	1.07	0.89–1.28
	2007	662	508	1170	57%	1.09	0.92–1.29
	2008	758	337	1095	69%	1.88	1.57–2.24
	2009	703	332	1035	68%	1.77	1.48–2.11
	2010	566	227	793	71%	2.08	1.71–2.53
Sex	Female	1924	1134	3058	63%	1.0	(reference)
	Male	1715	994	2709	63%	1.02	0.91–1.13
Facility	Primary care facility	2061	1488	3549	58%	1.0	(reference)
	Specialist Hospitals	1687	775	2462	69%	1.57	1.41–1.75
Urban facilities	Rural	954	936	1890	50%	1.0	(reference)
	Urban	2794	1327	4121	68%	2.07	1.85–2.31
Age at time of PCR	≤2 months	1257	599	1856	68%	1.0	(reference)
	>2 months	2491	1664	4155	60%	0.71	0.63–0.80

Factors associated with linkage of HIV PCR positive infants to antiretroviral therapy services among infants attending public sector health facilities in the Western Cape Province of South Africa, January 2005 to December 2010.

### Delays from PCR-positive Diagnosis to ART Services

**Figure 1:**
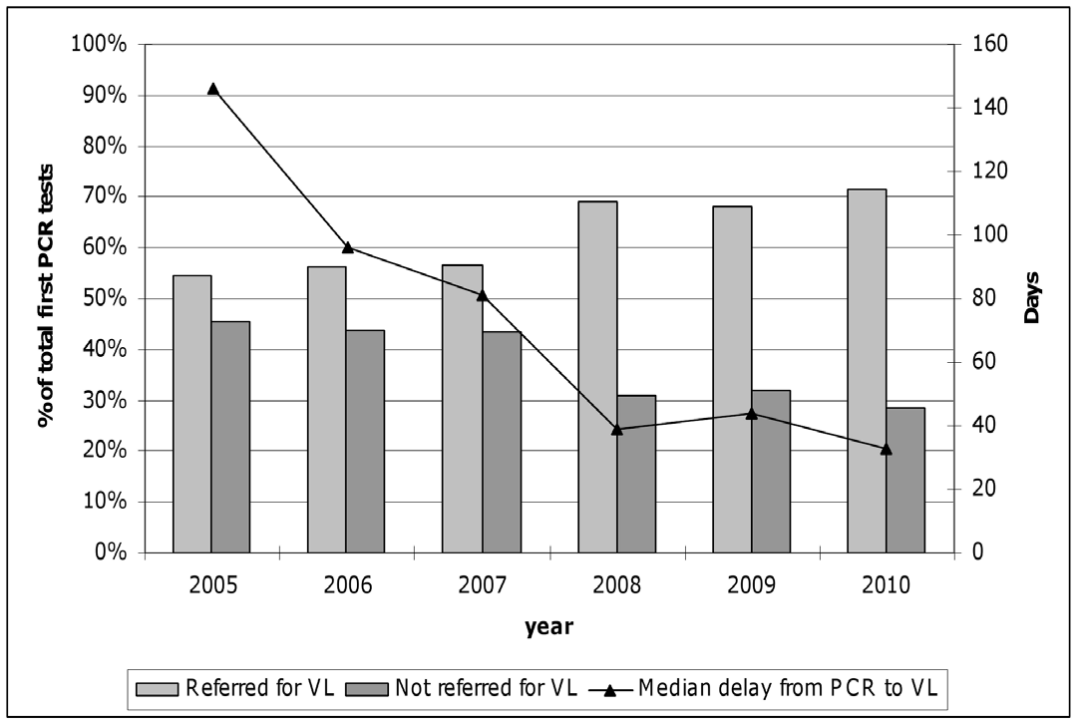
Linkage to antiretorivral therapy services. Proportion of infants testing positive on HIV PCR who are successfully linked to antiretroviral therapy services (as indicated by HIV viral load testing), with median delay between PCR and linked viral load (VL) testing.

The median delay between the first positive HIV PCR test and the first viral load conducted as part of ART work-up was 146 days in 2005 (IQR 42–500 days) and decreased to 33 days (IQR 8–83 days) during 2010 (Figure 1). The largest decrease was observed during the period 2007–2008, as the delay halved from 81 days in 2007 to 39 days in 2008. Overall, 66% and 85% of these delays were less than 150 days and 365 days, respectively. In 2010, 83% of delays were less than 150 days compared to the 50% during 2005.

The shortest delays in linkage of PCR-positive children to ART services were observed at specialist paediatric hospitals. The median delay of children at these hospitals was 13 days (IQR 5–68 days) compared to all other children who experienced a median delay of 87 days (IQR 28–287 days, p<0.001). The overall trend of reductions in delays over calendar years was similar for the two main specialist hospitals and all other facilities. The major reduction in delays during the 2007/2008 period was observed at both specialist hospitals and primary care centres.

**Figure 2:**
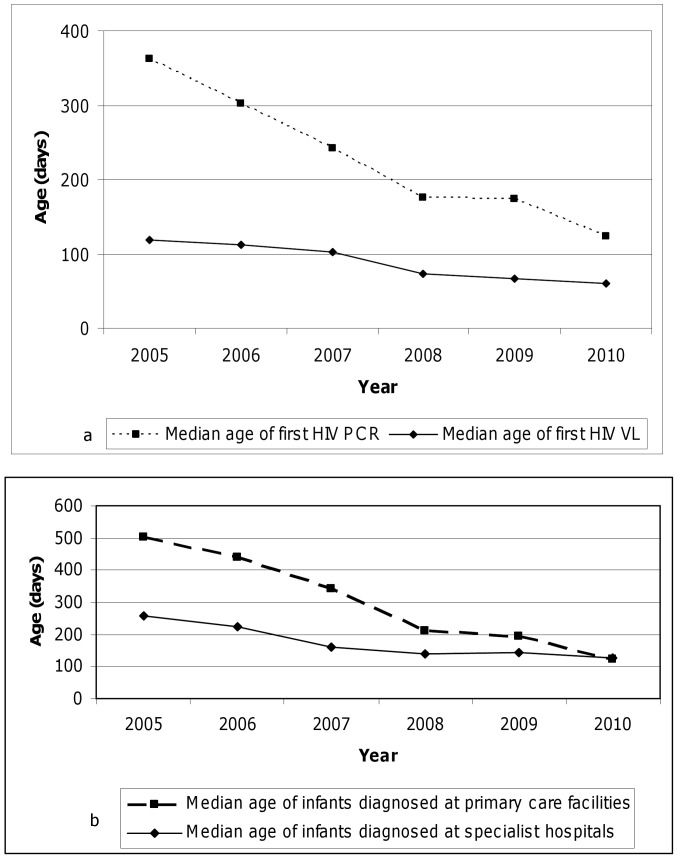
Age of children accessing early infant diagnosis and antiretroviral therapy services. 2a. The age children (in days) at the time of HIV PCR testing and attendance at antiretroviral therapy clinics (as indicated by HIV viral load testing), by year, among children attending public sector services in the Western Cape province, 2005–2010. The median age of first HIV viral load represent a small subset infants starting ART. This median age is mostly in the first 6 months due to the nature history of HIV disease progression in children. The median age of PCR reflects the age at which PCR testing are being used in all HIV exposed children, including much older children. 2b. The age children (in days) at the time of HIV viral load testing and attendance at antiretroviral therapy clinics, by year, among children attending public sector services in the Western Cape province, 2005–2010. The dotted line represents children diagnosed at primary care facility and the solid like represent children diagnosed at specialist facilities. The time to HIV diagnosis in primary care facilities had shown a great improvement over time, likely due to improved access to EID program. The diagnosis at specialist facilities represents children presenting with disease progression. In order to avoid HIV related paediatric mortality, the age of diagnosis at primary cares sites should be below the age of diagnosis at specialist hospitals.

The median age of PCR-positive children undergoing their first attendance at ART clinics, as indicated by pre-ART HIV viral load testing, was 96 days over the entire study period (IQR 50–169 days). This age decreased from 119 days in 2005 to 60 days in 2010, with 2007/2008 being the year of the most significant decrease (from 103 days in 2007 to 73 days in 2008) (Figure 2a). However, the reduction in median age of HIV viral load was mainly observed outside the two specialist hospitals (Figure 2b); the age of first HIV viral load in two hospitals remained constant over the study period.

## Discussion

These data demonstrate that only a fraction of children in this setting who test HIV PCR-positive within the PMTCT EID programme are successfully linked ART services. This proportion increased substantially during the period 2005–2010∶71% of the HIV infected infants in 2010 had a subsequent HIV viral load indicating their attendance at ART services. In parallel, among HIV-infected children who were successfully linked to ART services, the time delay between HIV PCR-positive test results and first attendance at ART services decreased in each successive year, though the median delay remained greater than 1 month during 2010.

Our data demonstrate that the number of infants tested doubled over the six year period, while the proportion of infants testing PCR positive declined from 12% to 3%. The reduction in infant HIV prevalence is seen in both asymptomatic children in the primary care setting and infants tested in hospitals. This steady decline points to the successes of the PMTCT programme in the Western Cape province, mirroring gains nationally [18], [19].

### Linkage of HIV-infected Infants to ART Services

There are several factors that may contribute to the failure of children testing HIV PCR positive in the EID programme to be linked to ART services. First, an infant could demise before the caregiver received the test result and/or attended the ART clinic. For example one Tanzanian study showed that 14% of caregivers received the EID result after their child had already died [12]. Second, even if a child is alive, HIV PCR test results may not be returned to caregivers. A previous study done in South Africa had shown that of 584 infants undergoing HIV PCR testing for EID at a routine immunization clinic, only 332 mothers (57%) returned to receive the results [13]. This type of loss to follow-up (LTF) is a major challenge, as it increases the risk of morbidity and mortality in infants due to untreated HIV infection [20].

Some of the phenomenon of LTF of mother-infant pairs is likely due to migration patterns of women during pregnancy and postpartum, as there is widespread anecdotal evidence of women from rural areas migrating to urban centres for antenatal care and delivery, and then returning to rural homes early postpartum. This form of ‘health migrancy’ has been documented in many parts of Africa [21], and presents a major challenge to continuity of care in maternal and child health. In the setting of our study this migration between health services may negatively bias our linkage to care estimate; the magnitude of this bias is difficult to estimate. An additional cause of phenomenon of LTF of mother-infant pairs operates at the level of health systems. In South Africa and elsewhere, EID testing is timed to coincide with the first infant immunization visit at 6 weeks of age, with results commonly returned to caregivers at the second immunization visit 10 weeks postpartum. Although this integration of EID into immunization services may reduce the burden of clinic visits for the caregiver, the 4-week interval between testing and receiving results may increase LTF and alternative systems for returning HIV PCR results need urgent consideration. For example, cellphone technology has been used to deliver EID results with some success in Zambia and warrants further investigation [22].

A third concern is LTF of caregiver/infant pairs who are diagnosed as HIV-positive but LTF before attending ART services. Prior to 2008 in this setting all HIV-infected infants were referred to specialist hospitals for ART care, and the time and cost of attending these hospital visits may present a barrier to many caregivers, particularly in rural areas [23]. Since 2008 there has been a shift towards paediatric ART services delivered through community-based primary care services, although since not all clinics offering EID also provide paediatric ART the separation of diagnostic and treatment services remains a barrier to rapid ART initiation in infants. In order to overcome these challenges in paediatric HIV care, a re-engineering of the current health systems to integrate the PMTCT, EID and infant HIV treatment is required. For instance, delivery of HIV specific services at immunization clinics instead of general paediatric clinics had been found to achieve superior uptake in Malawi [24].

### Delays in Attending ART Services

The time delay between positive HIV PCR test results and presentation to ART services decreased between 2005 and 2010 but remained more than 5 weeks in the most recent period. Several factors contribute to these delays. First, the number of primary care facilities that provide paediatric ART has increased during the past few years, reducing delays related to geographically distant referrals. Second, HIV testing for EID is based on PCR technology in central laboratories, creating delays around the transport of specimens, testing and return of results; thereafter, infants with positive test results may require referral to separate facilities for ART screening (including baseline HIV viral load testing) and initiation. In light of the steps required, a 4–5 week delay may approach the minimum possible delay under the current system.

The development of point-of-care EID assays based on HIV PCR [25] or HIV p24 antigen detection [26] may play a valuable role in further reducing these delays. Having access to the EID result on the same day using point-of-care testing could mean that appropriate counseling and clinical management can be initiated during the same clinic visit. This could have a positive impact on the proportion of children starting ART as well as the delay between diagnosis and treatment, and this possibility warrants further research.

### Strengths and Limitations

The interpretation of these data is subject to a number of limitations. While we included all HIV PCR tests conducted in public sector health facilities across the province, the coverage of the EID programme may not be complete, and the number of HIV-exposed infants who are not tested in the province is unknown. The EID coverage in low- and middle-income countries had been estimated to be around 15% [27]. However this is likely an underestimate for South Africa as more recent local data suggest the coverage in South Africa to be around 68% [1]. Second, we have used ‘baseline’ HIV viral load testing as a marker of attendance at ART services, which presumes that paediatric ART services adhere to policy guidelines and conduct viral load testing at an infant’s first ART clinic visit. While deviations from national guidelines for paediatric ART have not been documented, it is possible that some children were seen at ART clinics but do not have a viral load test; if this is the case, we may underestimate slightly the proportion of infected children who go on to start ART. It is also possible that children may access ART in the private sector and thus are not captured by our method, however private paediatric services account for a very small minority HIV related care in this setting, and thus this phenomenon is unlikely to impact our study findings. Third, as described above, there are multiple steps required for a child to be diagnosed as HIV PCR-positive and linked to ART services. Using laboratory data, we are unable to tell which specific barriers between EID and ART services are most important, and additional research is required to explore these in detail. Finally, these data should be generalized with caution, as the coverage of EID and ART services, and the strength of health systems more generally, varies widely between settings.

In summary, these data demonstrate that the proportion HIV-exposed infants testing PCR positive in the Western Cape province of South Africa has decreased dramatically since 2005. During the same period the proportion of HIV PCR-positive infants who subsequently attended ART services has increased substantially but remains suboptimal. While additional research is required to understand the barriers to successful ART referral, there is a clear need for interventions that facilitate linkage of infants diagnosed as HIV-infected to paediatric ART programmes. As the number of infant HIV infections decline, the absence of a direct system to trace HIV-infected infants and ‘fast track’ them onto ART is emerging as an important need in this setting as elsewhere in South Africa.
